# Vitamin K2 Can Rescue the Dexamethasone-Induced Downregulation of Osteoblast Autophagy and Mitophagy Thereby Restoring Osteoblast Function *In Vitro* and *In Vivo*

**DOI:** 10.3389/fphar.2020.01209

**Published:** 2020-08-11

**Authors:** Liang Chen, Xiang Shi, She-Ji Weng, Jun Xie, Jia-Hao Tang, De-Yi Yan, Bing-Zhang Wang, Zhong-Jie Xie, Zong-Yi Wu, Lei Yang

**Affiliations:** ^1^ Department of Orthopedic Surgery, The Second Affiliated Hospital and Yuying Children’s Hospital of Wenzhou Medical University, Wenzhou, China; ^2^ Key Laboratory of Orthopedics of Zhejiang Province, Wenzhou, China; ^3^ School of Mental Health, Wenzhou Medical University, Wenzhou, China

**Keywords:** Vitamin K2, dexamethasone, osteoblast, mitophagy, glucocorticoid-induced osteoporosis (GIOP)

## Abstract

Chronic long-term glucocorticoids (GC) use is associated with glucocorticoid-induced osteoporosis (GIOP) by inhibiting the survival and impairing the functions of osteoblasts. Autophagy and mitophagy play key roles in osteoblast differentiation, mineralization and survival, and mounting evidence have implicated osteoblast autophagy and mitophagy as a novel mechanism in the pathogenesis of GIOP. Vitamin K2 (VK2) is an essential nutrient supplement that have been shown to exert protective effects against osteoporotic bone loss including GIOP. In this study, we showed that the glucocorticoid dexamethasone (Dex) deregulated osteoblast autophagy and mitophagy by downregulating the expression of autophagic and mitophagic markers LC3-II, PINK1, Parkin. This consequently led to inhibition of osteoblast differentiation and mineralization function *in vitro*. Interestingly, co-treatment with VK2 significantly attenuated the Dex-induced downregulation of LC3-II, PINK1, Parkin, thereby restoring autophagic and mitophagic processes and normal osteoblastic activity. In addition, using an established rat model of GIOP, we showed that VK2 administration can protect rats against the deleterious effects of Dex on bone by reinstating autophagic and mitophagic activities in bone tissues. Collectively, our results provide new insights into the role of osteoblast autophagy and mitophagy in GIOP. Additionally, the use of VK2 supplementation to augment osteoblast autophagy/mitophagy may significantly improve clinical outcomes of GIOP patients.

## Introduction

Glucocorticoids (GC), are widely used clinically for the treatment of allergic and autoimmune diseases and cancer owing to their anti-inflammatory and immunosuppressive properties (Rizzoli and Biver, 2015). However, chronic or prolonged GC treatment exhibits detrimental effects on the skeleton causing rapid bone loss, progressive decrease in bone mineral density leading to bone fragility, often termed glucocorticoid-induced osteoporosis (GIOP) (Seibel et al., 2013; Hartmann et al., 2016). The molecular mechanism underlying GIOP is complex and has yet to be fully unraveled. This is because endogenous GC activities are necessary for the maintenance of bone homeostasis but excess GC impede bone formation by impairing osteoblast differentiation, mineralization function, and survival. The deleterious effects of GC on osteoblasts are now considered to play a major role in the development of GIOP (Weinstein et al., 1998; Canalis et al., 2007; Hartmann et al., 2016; Rizzoli, 2017).

Autophagy is a highly conserved homeostatic process that plays an essential part in the regulation of osteoblast differentiation and mineralization function (Liu et al., 2013; Nollet et al., 2014; Han et al., 2018; Lian et al., 2018). It has consistently been shown as a protective mechanism in maintaining osteoblast viability by allowing cells to survive various stresses (Todde et al., 2009; White and Lowe, 2009; Gu et al., 2016; Yang et al., 2016; Zhu et al., 2019; Zhao et al., 2020). Furthermore, GC usage have been reported to inhibit osteoblast autophagy and this effect plays a pivotal role in the pathogenesis of GIOP (Han et al., 2018; Lian et al., 2018; Gremminger et al., 2019; Wang et al., 2019). Recently a specialized form of autophagy that specifically degrades dysfunctional or redundant mitochondria, termed mitophagy, was found to contribute to the mineralization function of osteoblasts (Pei et al., 2018a). Under homeostatic conditions, PTEN induced putative kinase 1 (PINK1) translocates to the inner membrane of the mitochondria where it is rapidly degraded. When mitochondria are damaged (and becomes depolarized/loses membrane potential), PINK1 accumulates on the outer membrane where it phosphorylates ubiquitin and other mitochondrial outer membrane proteins, and facilitates the rapid recruitment of cytosolic Parkin, an E3 ubiquitin ligase, to the damaged mitochondria (Zhao et al., 2020). Multiple mitochondrial outer membrane proteins are ubiquitinated by Parkin resulting in the recruitment of ubiquitin-binding protein, p62/SQSTM1. The linkage of p62 to ubiquitin on the mitochondria and LC3-II (the lipidated form of LC3) on autophagosomes provides a physical attachment point for mitophagy (Geisler et al., 2010; Palikaras et al., 2018), and disruption of either PINK1 or Parkin leads to the impaired mitophagy (Han et al., 2015; Lazarou et al., 2015). Interestingly, impaired mitophagic function have been reported to negatively impact osteoblast differentiation and mineralization function *in vitro* (Shen et al., 2018b; Jing et al., 2020) and the restoration of mitophagy helps alleviate steriod-induced bone loss *in vivo* (Zhang et al., 2020). Despite these encouraging reports, the precise role of autophagy and mitophagy in osteoblastic differentiation and function has not been thoroughly explored.

A more complete understanding of the effects of GC on osteoblast autophagy/mitophagy will be paramount in the exploration of new therapeutic strategy for the treatment of GIOP. Vitamin K2 (VK2) is a fat-soluble vitamin that have been used clinically to treat osteoporotic bone loss. Various reports have further demonstrated that dietary VK2 supplementation is associated with improved bone formation status, reduced risk of GIOP, and lowered the prevalence of osteoporotic fracture (Sasaki et al., 2005; Iwamoto et al., 2010; Weng et al., 2019). Recent findings further indicate that the pro-osteoblastic effects of VK2 is mediated by enhanced autophagic activities (Li et al., 2019), but whether mitophagy is also associated with VK2’s beneficial effects in osteogenesis remains to be determined. In this study, we aim to investigate the potential beneficial effects of VK2 against the deleterious effects of GC (Dexamethasone; Dex) on osteoblasts particularly in terms of mitophagy and autophagy.

## Materials and Methods

### Reagents, Antibodies, and Media

Vitamin K2 (VK2), Dexamethasone (Dex), and 3-Methyladenine (3-MA) were purchased from Sigma-Aldrich (St. Louis, MO, USA). Primary antibodies against alpha-1 type I collagen (COL1A1), Runt-related transcription factor 2 (Runx2), and Osteocalcin (Ocn) were from Abcam (Cambridge, UK). The primary antibody against the mitochondrial import receptor subunit TOM20 was procured from Santa Cruz Biotechnology (Dallas, TX, USA). Primary antibodies against LC3-I/II, p62, PINK1, Parkin and GADPH were obtained from Cell Signaling Technology (Danver, MA, USA). Fetal bovine serum (FBS), Dulbecco’s Modified Eagle Medium (DMEM), and penicillin/streptomycin were purchased from Gibco BRL (Thermo Fisher Scientific; Waltham, MA, USA). Monodansylcadaverine (MDC) was acquired from Solarbio Science & Technology (Beijing, China). All other chemicals used were of analytical grade complying with tissue and cell culture standards.

### Cell Culture

Primary rat osteoblasts were isolated *via* sequential trypsin-collagenase digestion of excised calvarial bone from 1-day-old neonatal Sprague Dawley (SD) rats as previously described (Bakker and Klein-Nulend, 2012). Primary rat calvarial osteoblasts were cultured in complete DMEM (1% (v/v) penicillin–streptomycin and 10% (v/v) FBS) humidified conditions of 5% CO_2_ at 37°C. The medium was changed every other day and cells were passaged or used for downstream experiments upon reaching 80–90% confluence.

### Cytotoxicity Assay

The effect of Dex and VK2 on the viability of osteoblasts was determined using the Cell Counting Kit-8 (CCK-8) assay (MedChemExpress LLC; Monmouth Junction, NJ, USA). Primary calvarial osteoblasts seeded in 96-well plate at density of 5×10^3^ cells per well and divided into the following treatment groups: Dex (1, 5, 10, or 20 μM) for 12 and 24 h; VK2 (10^−8^, 10^−7^, 10^−6^, 10^−5^, or 10^−4^ M) for 24 and 48 h; and pre-treatment with Dex (10 μM) for 24 h then treated with VK2 (10^−6^ M) for further 48 h. Untreated cells were used as controls. At the end of the experimental period, 10 μl of CCK8 reagent was added to each well and for 2 further hours. The absorbance or optical density (OD) at wavelength of 450 nm were measured on a Multiskan Go Microplate Spectrophotometer (Thermo Fisher Scientific).

### Osteoblast Differentiation and Mineralization Assays

Primary calvarial osteoblasts were seeded at a density of 5×10^4^ cells per well in a 24-well plate. For the drug treatment, cells were pre-treated with 10 μM Dex for 24 h, then treated with 10^−6^ M VK2 without or with 5 mM 3-MA for 48 h as indicated. After treatment, cells were cultured under osteogenic conditions (complete DMEM containing 20 μM ascorbic acid and 10 mM β-glycerophosphate) with osteogenic media replaced every other day. Total cellular proteins were extracted on the 7^th^ day of osteogenesis for western blot analysis of proteins involved in osteoblast differentiation. Alkaline Phosphatase (ALP) activity was measured after 7 days of differentiation using the ALP Staining Kit (Beyotime Institute of Biotechnology; Jiangsu, China). For mineralization and bone nodule formation, cells were differentiated under osteogenic conditions for 21 days, fixed and then stained with Alizarin Red S (ARS) solution (Solarbio Science & Technology). The absorbance at 520 nm for ALP and at 570 nm for ARS staining was detected using a microplate reader.

### Western Blot Analyses

Total cell protein was extracted from cultured cells using RIPA lysis buffer (Beyotime Institute of Biotechnology) containing protease and phosphatase inhibitors (Sigma-Aldrich) for 30 min at 4°C. Cell lysates were cleared by centrifugation and protein concentration determined by the BCA Protein Assay Kit (Beyotime Institute of Biotechnology) as per manufacturer’s instruction. For each sample, 20 μg of extracted protein (diluted in SDS Sampling buffer and denatured by boiling for 5 min) were resolved on 10–15% SDS–polyacrylamide gel electrophoresis gel. Separated proteins then transferred to polyvinylidene difluoride (PVDF) membranes (Merck Millipore; Burlington, MA, USA) overnight at 4°C. PVDF membranes were blocked with 5% skim milk diluted in Tris buffered saline with 0.1% Tween 20 (TBST) for 2 h at room temperature and then incubated with indicated primary antibodies for 12 h at 4°C. After extensive washes with TBST, membranes were incubated with corresponding HRP-conjugated secondary antibodies for 4 h at room temperature. Proteins were visualized by enhanced chemiluminescence and imaged on a ChemiDoc XRS+ (Bio-Rad; Hercules, CA, USA). Protein bands were quantified by densitometry analysis using Image Lab V3.0 software (Bio-Rad).

### Immunofluorescence

After treatment period cells were fixed with 4% paraformaldehyde (PFA) for 15 min at room temperature and permeabilized with 0.5% (v/v) Triton X-100 in PBS for 20 min. Non-specific antibody binding was blocked with 1% (w/v) goat serum albumin for 1 h at room temperature and then incubated with indicated antibodies (the dilution for the antibodies is 1:200 respectively) in 0.2% BSA-PBS overnight at 4°C with gentle mechanical rocking. Cells were washed extensively and then incubated with fluorescence conjugated secondary antibodies (Alexa Fluor 488 or 546; Thermo Fisher Scientific) for 1 h at room temperature in the dark. Nuclei were stained with DAPI for 5 min at room temperature. Fluorescence images were captured on an Olympus BX53 fluorescence microscope (Olympus Life Science; Tokyo, Japan) and the level of expression was determined using integrated optical density (IOD) using Image-Pro Plus software (Media Cybernetics, Inc; Rockville, MD, USA). For MDC staining, primary calvarial osteoblasts cultured in 24-well plates and treated as indicated for 2 days were incubated with MDC reagent for 45 min at 37°C and fluorescence images captured as described earlier.

### Transmission Electron Microscopy (TEM)

Treated primary calvarial osteoblasts were fixed in 2.5% glutaraldehyde for 12 h at 4°C, post‐fixed in 2% osmium tetroxide for 1 h (Florencio-Silva et al., 2017), and then stained with 2% uranyl acetate for 1 h at room temperature. Cells were dehydrated in cold graded ethanol series (10 min each of: 30, 50, 70, 80, 90, and 100% ethanol) and then washed three times with 100% acetone (20 min each time with gentle rocking). Cells were then embedded in araldite epoxy resin, semi-thin sections were cut and stained with toluidine blue. TEM images were captured on a Hitachi Field Emission Transmission Electron Microscope (Hitachi High-Technologies Corp; Tokyo, Japan).

### Rat Model of Glucocorticoid-Induced Osteoporosis (GIOP)

All animal experiments were approved by the Animal Ethics Committee of The Second Affiliated Hospital and Yuying Children’s Hospital of Wenzhou Medical University, and conducted pursuant to the criteria outlined in the Guide for the Care and Use of Laboratory Animals (NIH, Bethesda, MD, USA). Forty 3-month-old SD male rats were purchased from Shanghai Laboratory Animal Center (SLACCAS; Shanghai, China) and housed in ventilated cages in groups of 5 under SPF conditions of 22–25°C with 12 h/day light duration. All animals had free access to tap water and standard rodent diet (containing 2.5% casein, 0.8% phosphorus, 1% calcium, 70–80% carbohydrates, and 5% fat) provided by Provimi Kliba, AG (Kaiseraugst, Switzerland). After one week of acclimatization, the rats were evenly and randomly divided into four groups (n = 10 per group): Sham (Vehicle) control group and three GIOP experimental groups. All rats in the GIOP groups received daily intraperitoneally injection of Dex (5mg/kg) for 4 weeks (Due to our results *in vitro* that high-dose and long-term use of Dex inhibited osteoblast mitophagy, we exerted the concentration of Dex of 5mg/kg/day to induce osteoblast mitophagy inhibiton *in vivo*). Rats in Control group received daily injections of PBS for the same period. After 4 weeks of Dex treatment, all rats in the GIOP groups were screened for osteoporotic bone loss by X-ray radiograph imaging and subsequently randomly assigned into the following treatment groups: Vehicle, VK2 (30 mg/kg), and VK2 + 3-MA (15 mg/kg). 3-MA is a widely acknowledged autophagy inhibitor. The aim of 3-MA administration is to block the pro-autophagic effects of VK2. VK2 and 3-MA were injected intraperitoneally daily for 8 weeks after which all rats were euthanized. Bilateral femurs were excised, cleaned of soft tissues and then fixed in 4% PFA prior to micro-computed tomography (CT) and histological assessment.

### Micro-CT Analysis

Microstructural analysis of the distal femoral bones was carried out on a cabinet cone-beam micro-CT system and associated software (μCT 50, Scanco Medical; Brüttisellen, Switzerland). Images were acquired at a voltage of 70 kV, electric current of 200 μA and a spatial resolution of 14.8 mm in all directions. Three-dimensional reconstructed images were generated and the volume of interest (VOI) analyzed included the trabecular compartment 2 mm below the highest point of the growth plate to distal 100 CT slices. Quantitative bone parameters were assessed within the VOI included percentage bone volume to tissue volume (BV/TV), the mean trabecular thickness (Tb.Th, mm), the mean trabecular number (Tb.N, 1/mm), the mean trabecular separation (mean width of the medullary cavity between trabeculae, Tb.Sp, mm) using Evaluation V6.5-3 in micro-CT system and associated software.

### Histology, Immunohistochemistry and Immunofluorescence Staining

For histological and immunohistochemical (IHC) assessments, fixed femoral bone tissues were decalcified in 10% EDTA for 3 weeks, dehydrated in graded ethanol series (70 to 100%), cleared in xylene, and paraffin-embedded with the long axis of the bone parallel to the base plane to preserve anatomical orientation. Longitudinal serial sections of 4 μm thick were cut and mounted on poly-lysine coated microscope slides and then subjected to hematoxylin and eosin (H&E), and Masson’s trichrome staining as per standard laboratory protocols. For IHC staining, 6 μm sections were prepared and incubated with primary antibodies against LC3-II, Parkin, and OCN (the dilution for the antibodies is 1:200 respectively) and the immuno-reactivities in the sections were detected using a horseradish peroxidase detection system in accordance with manufacturer’s protocol (Vector Laboratories; Burlingame, CA, USA). For immunofluorescence staining, 6 μm thick sections were deparaffinized, rehydrated and then stained with primary antibody against LC3-II (the dilution for the LC3-II is 1:200) overnight at 4°C and then with secondary fluorescence antibody for 1 hour at room temperature in the dark. Nuclei were counterstained with DAPI for 5 min. Tissue sections were imaged under an Olympus BX53 light/fluorescence microscope equipped with a Olympus DP71 digital color camera (Olympus Life Science). The level of expression was determined using integrated optical density (IOD) using Image-Pro Plus software (Media Cybernetics, Inc; Rockville, MD, USA).

### Statistical Analyses

All statistical analyses were performed using the GraphPad Prism software (San Diego, CA, USA) and data presented herein are expressed as the mean ± standard error of mean (SEM) from at least three experimental repeats. Two tailed Student’s t-test was used to compare means between two groups and one-way ANOVA with Bonferroni or Dunnett corrections for multiple comparisons where appropriate. Differences were determined to be statistically significant when p < 0.05 unless otherwise stated.

## Results

### Dexamethasone (Dex) Exhibits Dose- and Time-Dependent Effects on Osteoblast Autophagy and Mitophagy

**Figure 1:**
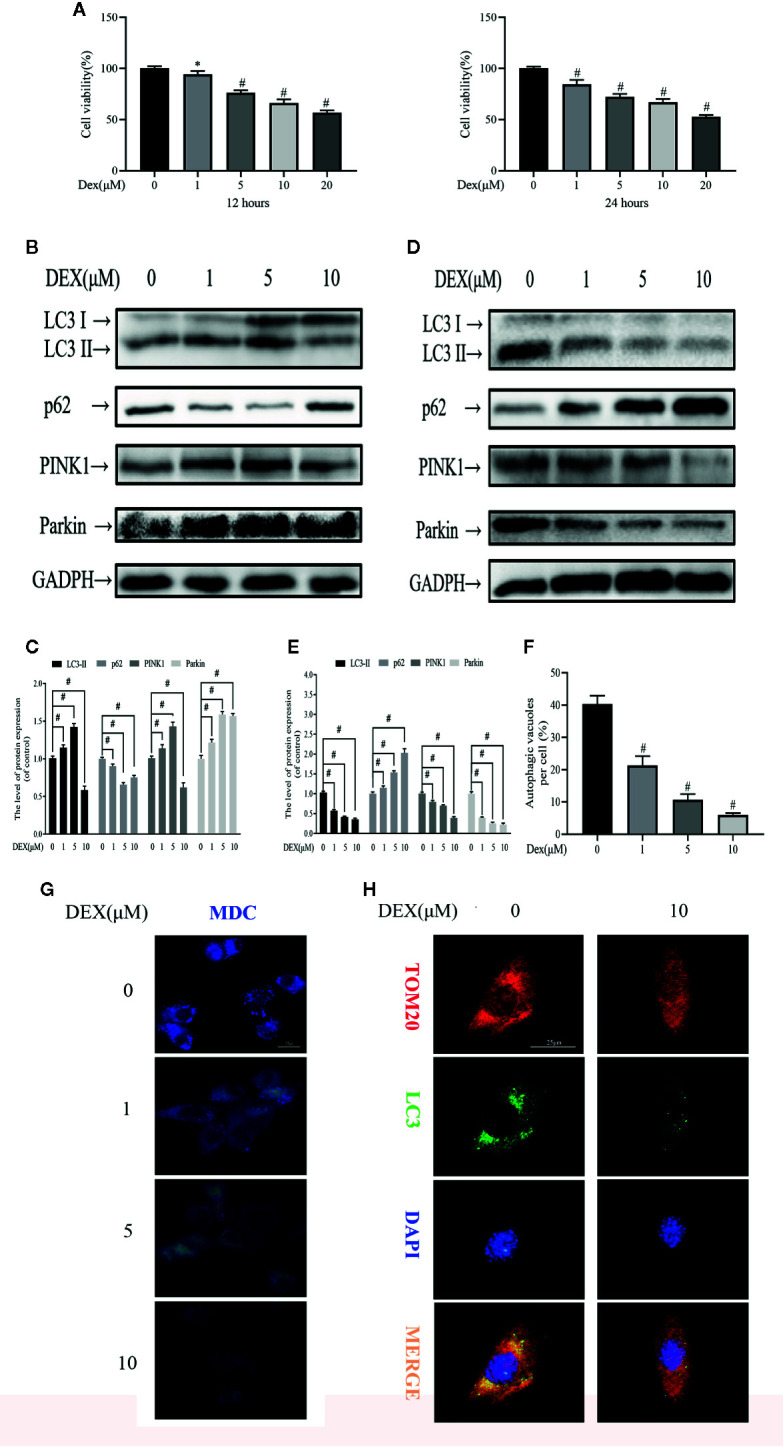
Dexamethasone (Dex) exhibits dose- and time-dependent effects on osteoblast autophagy and mitophagy. **(A)** Effects of different concentrations of Dexamethasone on the growth of osteoblasts after being treated for 12 h or 24 h, as measured by CCK8 assays; Western blotting results showed the change of mitophagy-related protein expression levels after 12 h **(B)** or 24 h **(D)** treatment; **(C, E)** Analysis of western blot results of **(B, D)**; **(F, G)** MDC is a marker for autolysosomes. MDC staining was used to confirm the alteration of the abundance of autophagic vacuoles in aforementioned group; **(H)** Representative images of immunofluorescence double staining of LC3 and Tom20 in osteoblasts. Data was expressed as mean ± SEM, n = 5; *p < 0.05, ^#^p < 0.01 vs. control group.

We first assessed the biological effects of Dex on osteoblast viability using the CCK-8 assay. As shown in the **Figure 1A**, Dex exhibited an inhibitory effect to osteoblast viability in a dose-dependent manner at 12 and 24 h after treatment, with an IC50 of about 20 μM (p<0.01). Next, we used western blot to analyze the expression of proteins involved in autophagy and mitophagy in Dex-treated osteoblasts. To our surprise, we found that low concentrations of Dex treatment (1 and 5 μM) for 12 h upregulated the expression of autophagy and mitophagy related proteins LC3-II, PINK1 and Parkin (p<0.01). This was also associated with a concomitant reduction in the expression of p62 (**Figures 1B, C**). On the other hand, treatment with 10 μM Dex potently inhibited the expression of LC3-II, PINK1 and Parkin. At 24 h Dex treatment, the expression of LC3-II, PINK1, and Parkin were all markedly reduced (p<0.01) (**Figures 1D, E**). We further explored the inhibitory effect of Dex on autophagy by staining cells with MDC, a fluorescent marker that accumulates in autophagic vacuoles. As shown in **Figure 1F**, the number of MDC-stained autophagic vacuoles were dose-dependently reduced following treatment with Dex for 24 h (**Figures 1F, G**). Furthermore, extensive colocalization of TOM20, a mitochondrial outer membrane protein, with LC3-stained autophagosomes in untreated osteoblasts was observed indicative of active mitophagy (**Figure 1H**). Treatment with 10 μM Dex for 24 h significantly attenuated the expression of LC3 indicating the inhibition of autophagosome formation and consequently impairment of mitophagy.

### Vitamin K2 (VK2) Activates Both Autophagy and Mitophagy in Osteoblasts

**Figure 2:**
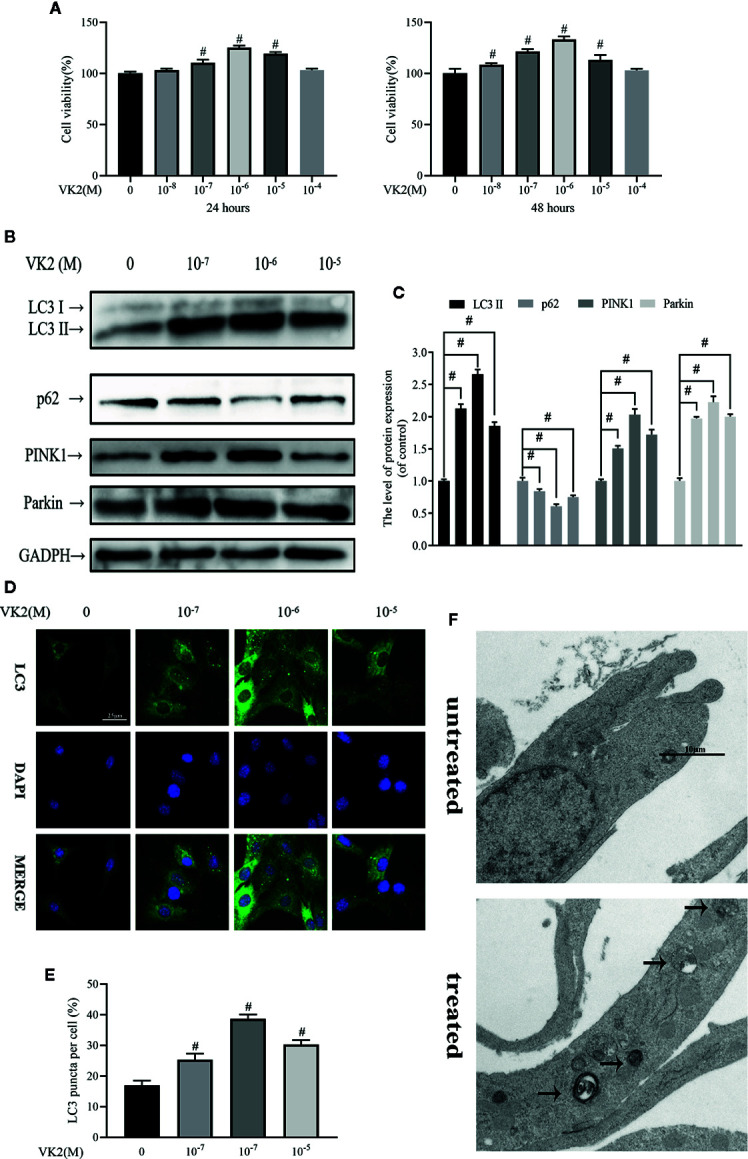
Vitamin K2 (VK2) activates both autophagy and mitophagy in osteoblasts. **(A)** Effects of different concentrations of Vitamin K2 on the growth of osteoblasts after being treated for 24 h or 48 h, as measured by CCK8 assays; **(B)** Western blot results showed the change of mitophagy-related protein expression; **(C)** Analysis of the western blotting results in **(B)**; **(D, E)** Representative images and quantified analysis of immunofluorescence staining of LC3 in osteoblasts; **(F)** Mitophagosomes were detected by transmission electron microscopy (×10000) in osteoblasts. (Black arrow: mitophagosome). Data was expressed as mean ± SEM, n = 5; ^#^p < 0.01 vs. control group.

As with Dex, we first examined potential cytotoxic effects of VK2 using the CCK-8 assay. Treatment with increasing concentrations of VK2 (10^−8^ to 10^−4^ M) did not induce any deleterious effects on primary osteoblast viability at 24 and 48 h after treatment (**Figure 2A**). In fact, cell proliferation was elevated when cells were treated with 10^−7^ to 10^−5^ M at 24 h and with 10^−8^ to 10^−5^ M at 48 h. Having established that VK2 has no adverse effect cell viability/proliferation, we next examined whether VK2 can induced autophagy/mitophagy in treated primary osteoblasts. As shown in **Figures 2B, C**, treatment of primary osteoblasts with VK2 for 48 h upregulated the expression of LC3-II, PINK1 and Parkin. The expression of p62 was also accordingly decreased (p<0.01). The induction of autophagy by VK2 was also confirmed by immunofluorescence staining for LC3 autophagosomes with the most potent inductive effect at the concentration of 10^−6^ M for 48 h (**Figures 2D, E**). This was further corroborated by TEM analysis showing increased formation of double-membraned vesicles in treated osteoblasts (**Figure 2F**). Collectively, VK2 treatment induces autophagy/mitophagy in primary calvarial osteoblasts and based on these results VK2 at concentration of 10^−6^ M was used for subsequent experiments.

### VK2 Alleviates Inhibitory Effects of Dex on Osteoblast Autophagy and Mitophagy

**Figure 3:**
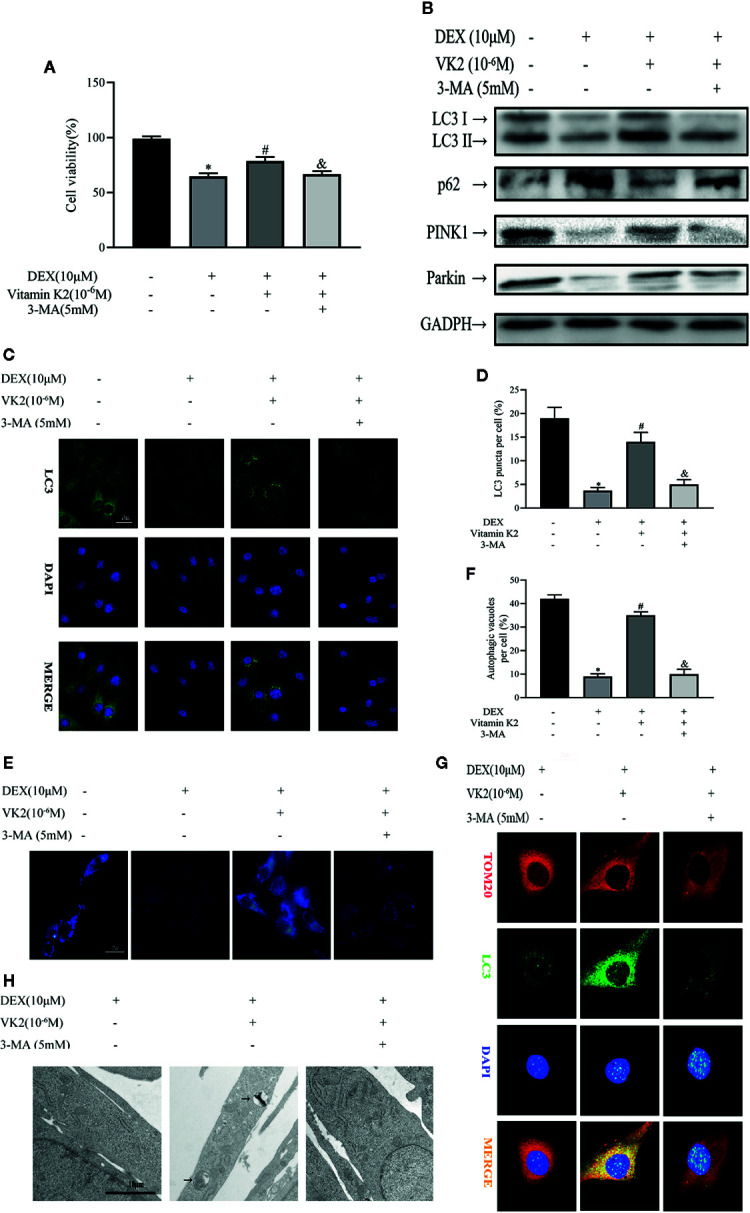
VK2 alleviates inhibitory effects of Dex on osteoblast autophagy and mitophagy. Primary osteoblasts were initially treated with 10 μM Dex for 24 h, then 10^−6^ M VK2 without/with 5 mM 3-MA was added into the culture medium for another 48 h. **(A)** Effects of Vitamin K2 on the growth of osteoblasts under the inhibition of Dexamethasone with/without 3-MA, as measured by CCK8 assays; **(B)** Western blot results showed the alteration of mitophagy-related protein expression levels of the above indicators; **(C, D)** Representative images and quantified analysis of immunofluorescence staining of LC3 in osteoblasts; **(E, F)** MDC staining was used to confirm the alteration of the abundance of autophagic vacuoles in aforementioned group; **(G)** Representative images of immunofluorescence double staining of LC3 and Tom20 in osteoblasts; **(H)** Mitophagosomes were detected by transmission electron microscopy (×10,000) in osteoblasts. (Black arrow: mitophagosome). Data was expressed as mean ± SEM, n = 5; *p < 0.01 vs. control group, ^#^p < 0.01 vs. DEX group, ^&^p < 0.01 vs. DEX+VK2 group.

Given the stimulatory effect of VK2 on osteoblast mitophagy, we next explored the possibility for VK2 in antagonizing the inhibitory effect of Dex against autophagic/mitophagic activities. To this end, primary osteoblasts were initially treated with 10 μM Dex for 24 h, then 10^−6^ M VK2 without/with 5 mM 3-MA was added into the culture medium for another 48 h. The 3-MA is a widely acknowledged inhibitor of autophagy and thus used to reverse the pro-autophagic/mitophagic effects of VK2. As shown in **Figure 3A**, 10^−6^ M VK2 can protect cells from the deleterious effects of 10 μM Dex on cell viability (p<0.01), whereas the addition of 3-MA partly attenuated the protective effects of VK2 treatment (p<0.01). Additionally, co-treatment of VK2 as indicated attenuated Dex-induced downregulation of LC3-II, PINK1, and Parkin protein expression (**Figure 3B**). Consistently, the upregulation of p62 following Dex treatment was also abrogated following co-treatment with VK2 treatment as well as the inhibition of autophagosome formation (**Figures 3C–F**). Immunofluorescence of co-localization of LC3 and TOM20 and TEM analysis further showed that treatment of cells with VK2 can alleviate the inhibitory effect of Dex on autophagosome formation mitophagy (**Figures 3G, H**). The co-treatment of 5 mM 3-MA potently blocked the upregulation of LC3-II, PINK1, and Parkin (**Figure 3B**), and subsequent induction of autophagy and mitophagy by VK2. Together, the above results indicate that VK2 can alleviate the inhibitory effect of Dex on osteoblast autophagy/mitophagy by preventing the downregulation of LC3-II, PINK1 and Parkin protein expressions.

### Autophagy and Mitophagy Are Both Necessary for Osteoblast Differentiation and Mineralization

**Figure 4:**
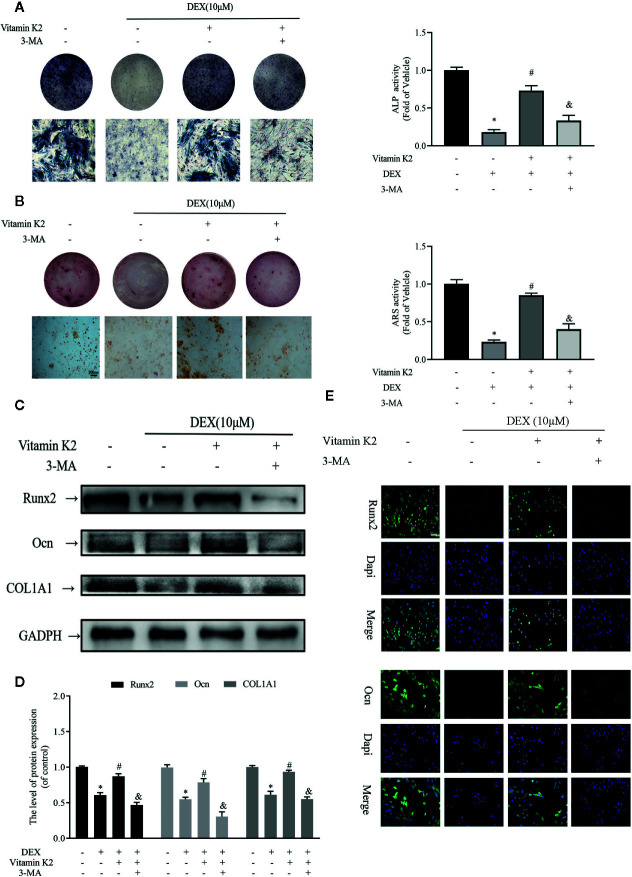
Autophagy and mitophagy are both necessary for osteoblast differentiation and mineralization. Primary osteoblasts were initially treated with 10 μM Dex for 24 h, then 10^−6^ M VK2 without/with 5 mM 3-MA was added into the culture medium for another 48 h. After treatment, the osteoblasts were cultured in osteogenic medium for indicated days. **(A)** Osteoblastic functions were detected by ALP staining on the 7th day; **(B)** and mineralization degree by the ARS staining on the 21st day; **(C)** The osteogenic protein expression levels of the above indicators were detected by western-blotting and **(E)** the analysis of the results; **(D)** Representative immunofluorescence images of the above indicators, counterstained with Dapi. Data was expressed as mean ± SEM, n = 5; *p < 0.01 vs. control group, ^#^p < 0.01 vs. DEX group, ^&^p< 0.01 vs. DEX+VK2 group.

Next the effects Dex and VK2 against the osteogenic differentiation of osteoblasts were evaluated in our study. As shown in **Figures 4A, B**, compared with untreated controls, Dex treatment significantly reduced osteoblast differentiation (**Figure 4A**) and mineralization function (**Figure 4B**) respectively (p<0.01). On the other hand, treatment with VK2 countered the inhibition of Dex on osteoblast differentiation and mineralization (p<0.01). The positive effect of VK2 against Dex was further corroborated by western blot and immunofluorescence analysis of osteoblast marker protein expression. Consistent with cellular effects, the downregulation of Runx2, Ocn, and COL1A1 by Dex can be prevented with treatment with VK2 (**Figures 4C–E**, p<0.01). However, the pro-osteoblastic effects of VK2 against Dex can be further abolished with treatment with 3-MA. Thus, the results presented here, shows that VK2 can counter the anti-osteoblastic effects of Dex and that functional autophagic/mitophagic activity is necessary for efficient osteoblast differentiation and mineralization function.

### Protective Effects of VK2 Against Dex-Induced Bone Loss (GIOP) in Rats

**Figure 5:**
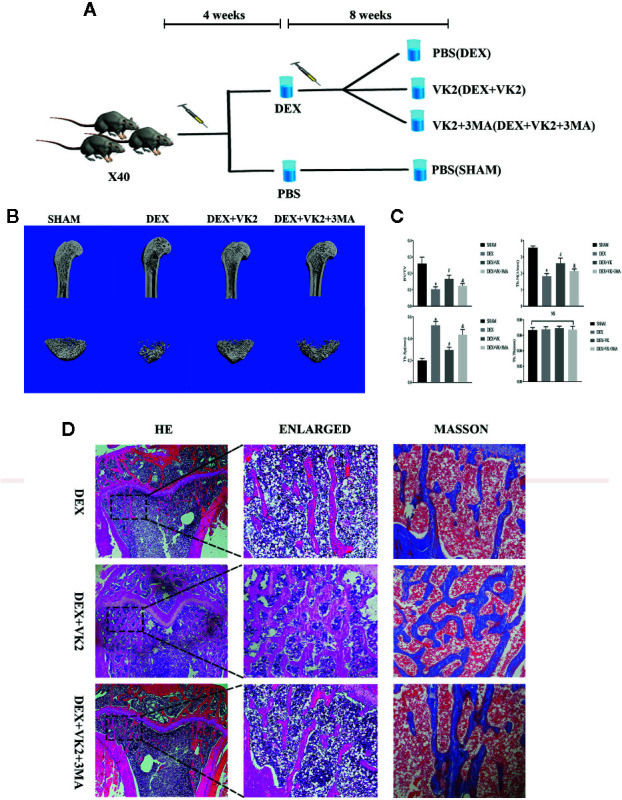
VK2 against Dex-induced bone loss (GIOP) in rats. **(A)** Schematic of experimental animal arrangement in this study; **(B)** Micro-CT images of the longitudinal sections of the distal femurs were taken; **(C)** Quantitative analysis results of BV/TV, Tb.N, Tb.Sp, and Tb.Th were divided into four groups; **(D)** H&E staining and Masson’s staining of metaphyseal tissue sections of femurs. Data was expressed as mean ± SEM, *p < 0.01 vs SHAM group, ^#^p < 0.01 vs. DEX group, ^&^p < 0.01 vs. DEX+VK2 group.

We next assessed whether the protective effects of VK2 against the deleterious effect of Dex *in vitro* can be recapitulated *in vivo* using the rat GIOP model. SD rats were treated with Dex for 4 weeks to induce bone loss and then administered with VK2 or VK2 + 3-MA for further 8 weeks (**Figure 5A**). Three-dimensional reconstructions of micro-CT scans showed extensive trabecular bone loss in the distal femur following Dex administration as compared to Sham controls (**Figure 5B**). This was confirmed by morphometric analyses which showed marked reduction in bone volume (BV/TV) and trabecular number, increased trabecular spacing, but no change in trabecular thickness (**Figure 5C**, p<0.01). On the other hand, rats that were administered VK2 for 8 weeks after Dex treatment showed significant restoration in trabecular bone architecture (**Figure 5B**), as well as improvements in BV/TV, Tb.N, and Tb.Sp. The protective effect of VK2 against Dex-induced trabecular bone loss was further confirmed from histological H&E and Masson trichrome staining of femoral bone tissues (**Figure 5D**). Co-treatment with 3-MA, blocked VK2 protective effect against Dex-induced bone loss suggesting that autophagy/mitophagy is in part accountable for mediating a guardian role of VK2.

**Figure 6:**
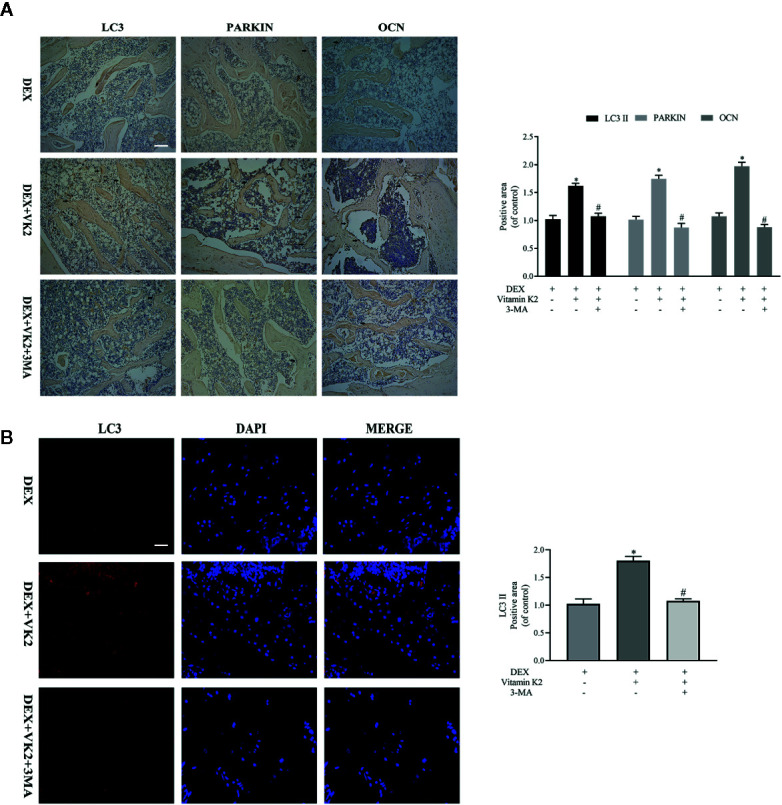
VK2 restored mitophagy and revived function of osteoblasts *in vivo*. The expression of the proteins in the femurs of the rats were further detected by **(A)** Immunohistochemical staining and quantitative analysis (black arrows: positive area); **(B)** Immunofluorescence and quantitative analysis. Data was expressed as mean ± SEM, *p < 0.01 vs DEX group, ^#^p < 0.01 vs. DEX+VK2 group.

To show that restoration of autophagy/mitophagy was indeed involved in mediating the protective effects of VK2 *in vivo*, we carried out IHC and immunofluorescence staining of femoral bone tissues from each treatment group. Compared with Dex treatment only, administration of VK2 following 8-weeks of Dex treatment significantly elevated the expression LC3 and Parkin in bone tissues indicating the restoration of active autophagic/mitophagic activities in osteoblastic cells (**Figures 6A, B**, p<0.01). In contrast, co-treatment with 3-MA, abolished the autophagy/mitophagy inducing effects of VK2 thereby preventing the restoration of bone architecture following Dex treatment.

## Discussion

**Figure 7:**
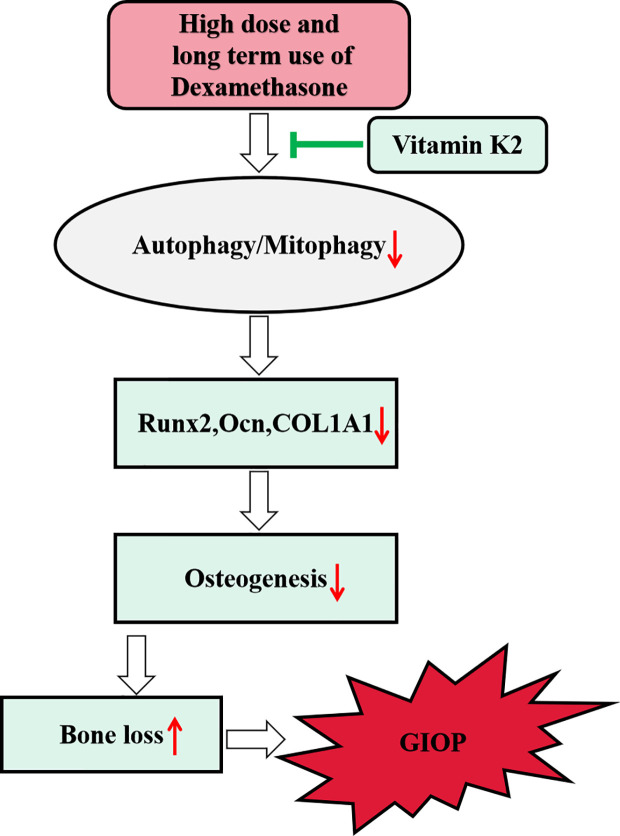
Schematic diagram of proposed mechanism underlying the role of autophagy/mitophagy in mediating the beneficial effect of VK2 against Dexamethasone-induced osteoporosis.

In the present study, we showed high dose of Dex treatment markedly attenuated osteoblast autophagy and mitophagy (the selective degradation of damaged mitochondria by autophagy) *via* the downregulation of LC3-II, PINK1, and Parkin protein expression, and the upregulation of p62. Consequently, this led to decreased osteoblast survival, inhibition of osteoblast differentiation and mineralization activity *in vitro*, and induced bone loss in rat model of GIOP. Interestingly, treatment with vitamin K2 (VK2) restored autophagic/mitophagic activities in Dex-treated osteoblasts, and reversed the deleterious effects of Dex on osteoblast survival, differentiation, and mineralization activity *in vitro*. Furthermore, administration of VK2 for 8 weeks following GIOP, protected rats from further bone deterioration and restored bone volume and bone microarchitecture integrity. Preliminary insights into the underlying mechanism suggests that VK2 treatment rescues osteoblastic function in response to Dex *via* restoring autophagic/mitophagic responses. Schematics of our study have been shown in **Figure 7**.

Although the underlying regulatory mechanism(s) of GIOP are unclear, it has been suggested that excess GCs may impeded osteoblast survival and bone formation activity through multiple pathways (Dalle Carbonare et al., 2001; Hartmann et al., 2016). In particular, the process of autophagy has gained much attention over the years as a potential driving mechanism in the development of GIOP (Lian et al., 2018; Shen et al., 2018a). Autophagy is a fundamental cellular degradative process that functions to remove or recycles dysfunctional protein aggregates and damaged organelles to maintain regular physiological activity (Klionsky and Emr, 2000; White and Lowe, 2009). However, when the autophagic process is out of control, that is, either too much or too little, apoptosis can be triggered leading to cell death (Minina et al., 2014). Meanwhile, mitophagy is an organelle-specific autophagic process whereby damaged or effete mitochondria are delivered to autophagosomes for degradation. It is one of the major mitochondrial quality control process that maintains optimal mitochondrial integrity. It is well known that GC excess interrupts mitochondrial function and prolonged exposure deteriorates mitochondrial integrity leading to release of pro-apoptotic proteins, reactive oxygen species (ROS) accumulation, and ineffective generation of ATP that markedly impedes osteoblast survival. Recent studies have suggested that mitophagy is important in regulating osteoblast survival and function (Pei et al., 2018a; Zhao et al., 2020). Nevertheless, the involvement of osteoblast mitophagy in the pathogenesis of GIOP is not clear. The present study showed that high-dose Dex treatment inhibited osteoblast autophagy and mitophagy by markedly downregulating the expression of LC3-II, PINK1 and Parkin. The reduction in the expression of key autophagic and mitophagic proteins impairs the induction of autophagy and mitophagy which is associated with hampered osteoblast differentiation and bone formation function *in vitro* and *in vivo*.

VK2 has attracted much attention over the years as an auxiliary drug for preventing and treating osteoporotic bone loss (Palermo et al., 2017). In fact, epidemiological studies have found that a lack of VK2 supplement is associated with a higher risk of osteoporosis and osteoarthritis in older individuals (Hauschka et al., 1975). Clinical administration of VK2 has been found to potently increase serum osteocalcin and lower the incidence of fractures in osteoporotic patients (Shiraki et al., 2000; Knapen et al., 2007). Additionally, VK2 administration was also found to protect against GC-induced bone loss in patients undergoing GC therapy (Inoue et al., 2001; Sasaki et al., 2005) and in animal models (Hara et al., 1993). Accordingly, Zhang et al. (2016) has proved that supplement with VK2 could prevent bone marrow stem cells from methylprednisolone-induced osteogenic malfunction and apoptosis. VK2 likely exerts its protective effects by enhancing osteoblast differentiation and mineralization function (Atkins et al., 2009; Rubinacci, 2009). In our current study, we found out this rescue event is associated with the activation of osteoblastic autophagy and mitophagy. We observed that co-treatment with VK2 potently enhanced osteoblast autophagy and mitophagy. Correspondingly, the deleterious effects of Dex on osteoblast differentiation and function can be ameliorated by treatment with VK2. VK2 was found to reinstate the expression of LC3-II, PINK1 and Parkin, thereby restoring osteoblast autophagy and mitophagy. The positive effects of VK2 on autophagy is consistent with previous report (Li et al., 2019). To further explore the beneficial roles of VK2, 3-MA, a potent autophagy inhibitor, was used in our study. Consistent with our findings, the administration of 3-MA partly abolished the effects of VK2 both in osteoblasts *in vitro* and *in vivo*.

Although there are several novel findings in this study, one question that the underlying mechanism of VK2 influence on osteoblast autophagy/mitophagy is still unclear. AMP-activated protein kinase (AMPK), as a critical sensor of cellular energy balance, is reported be crucial in cellular homeostasis (Pei et al., 2018b). Recent studies have revealed that AMPK phosphorylation promotes autophagy, as well as mitophagy, and plays important parts in mitochondrial quality control (Herzig and Shaw, 2018). In addition, several studies have reported that AMPK pathway activation is associated with the positive effects of VK2 (Bordoloi et al., 2019; Duan et al., 2020). Furthermore, Su et al. (2020) has demonstrated that VK2 treatment improves mitochondrial function in skeletal muscle *via* activating AMPK-SIRT1 pathway. Therefore, our preliminary insights suggest that AMPK pathway is responsible for the beneficial effects of VK2 in autophagy/mitophagy activation. Hence, signal pathways including AMPK should be investigated to elucidate the function of VK2 in the future studies.

## Conclusion

In summary, we have provided evidence that VK2 can protect osteoblasts against the deleterious effects of Dex *in vitro* and *in vivo* through the restoration of osteoblastic autophagic and mitophagic activities. However, a potential limitation of our study is that we only evaluated the effects of VK2 during the early-mid phases of GIOP, whereas clinically in patients, corticosteroid treatment is usually taken for life. Thus future studies will need to examine the long term effects of VK2 against GIOP. Despite this limitation, our study has shown that osteoblast autophagy and mitophagy are crucial cellular process needed for the osteoblast differentiation and mineralization function. These results provide new insights into the role of osteoblast autophagy and mitophagy in GIOP, and the maintenance of osteoblastic autophagy using VK2 supplementation may significantly improve clinical outcomes of GIOP patients.

## Data Availability Statement 

The raw data supporting the conclusions of this article will be made available by the authors, without undue reservation.

## Ethics Statement

The animal study was reviewed and approved by Animal Care and Use Committee of Wenzhou Medical University, China.

## Author Contributions

All the listed authors made substantial contributions to the study. LY, LC, JX, and D-YY participated in the experimental design and contributed reagents, materials, and analytical tools. XS, S-JW, J-HT, Z-JX, and Z-YW were also involved in the experiment. LY and LC wrote the manuscript. LC, JX, D-YY, S-JW, J-HT, and B-ZW are involved in data analysis. All authors contributed to the article and approved the submitted version. All the data shown in the figure are from the above authors’ experiments and can be used.

## Funding

This work was funded by a research grant to Science and Technology Department of Zhejiang Province (Grant No.:2016C37122) and National Natural Science Foundation of China (Grant No.:81772348).

## Conflict of Interest

The authors declare that the research was conducted in the absence of any commercial or financial relationships that could be construed as a potential conflict of interest.
